# Photoprotection of Cerium Oxide Nanoparticles against UVA radiation-induced Senescence of Human Skin Fibroblasts due to their Antioxidant Properties

**DOI:** 10.1038/s41598-019-39486-7

**Published:** 2019-02-22

**Authors:** Yaxi Li, Xiaoyang Hou, Chunsheng Yang, Yanyu Pang, Xinxin Li, Guan Jiang, Yanqun Liu

**Affiliations:** 1 Department of Dermatology, Affiliated Hospital of Xuzhou Medical University, Xuzhou, 221002 China; 2 Department of Dermatology, the Affiliated Huai’an Hospital of Xuzhou Medical University, the Second People’s Hospital of Huai’an, Huai’an, 223002 China

## Abstract

Ultraviolet (UV) irradiation, particularly ultraviolet A (UVA), stimulates reactive oxygen species (ROS) production in the epidermis and dermis, which plays a major part in the photoageing of human skin. Several studies have demonstrated that cerium oxide nanoparticles (CeO_2_ NP) can exhibit an antioxidant effect and free radical scavenging activity. However, the protective role of CeO_2_ NP in skin photoageing and the underlying mechanisms are unclear. In this study, we investigated the effects of CeO_2_ NP on UVA-irradiated human skin fibroblasts (HSFs) and explored the potential signalling pathway. CeO_2_ NP had no apparent cytotoxicity, and could reduce the production of proinflammatory cytokines, intracellular ROS, senescence-associated β-galactosidase activity, and downregulate phosphorylation of c-Jun N-terminal kinases (JNKs) after exposure to UVA radiation. Based on our findings, CeO_2_ NPs have great potential against UVA radiation-induced photoageing in HSFs via regulating the JNK signal-transduction pathway to inhibit oxidative stress and DNA damage.

## Introduction

Skin ageing is caused by environmental aggressors and the passage of time, and is one of the most common dermatologic concerns^1,2^. It is classified into two types—“intrinsic ageing” (attributed to the influence of genes and hormones) and “extrinsic ageing” (induced by environmental factors such as cigarette smoking, poor nutrition and solar radiation)^3,4^. Extrinsic ageing is mainly “photoageing”, which is caused by a combination of wavelengths of light, such as the visible spectrum, infrared radiation and ultraviolet (UV) radiation. Photoageing occurs with the ageing of epidermal and dermal cells. Senescent cells can, in general, be recognized by changes in morphology, high levels of reactive oxygen species (ROS) and enhanced activity of senescence-associated β-galactosidase (SA-β-gal) in lysosomal systems^5–7^. Increased release of secretory proteins such as interleukins (ILs), chemokines and growth factors is another hallmark of senescent cells^5,8^. Among the ILs secreted by senescent cells, IL-6, IL-8 and IL-1βare the most important^9^.

At present, UV radiation in sunlight is recognized as the important factor that induces photoageing, particularly ultraviolet A (UVA, 320–400 nm) radiation. UVA accounts for >95% of solar UV radiation and is present in sunlight all day. It can penetrate deep into the epidermis and dermis to cause photo-carcinogenesis, and has a critical role in photoageing^10^. Inflammation and overexpressed ROS are the two major pathogeneses of skin photoageing^11^. Inflammatory factors and high production of ROS can induce lipid peroxidation and oxidation of proteins and carbohydrates, as well as their accumulation in the dermis and epidermis of photo-damaged skin.

In the past decade, a growing number of nanomaterials have been implemented for biomedical areas, such as the magnetic resonance imaging (MRI), contrast media, drug carriers, and nanometer catalysts^12^. Cerium oxide nanoparticles (CeO_2_ NP) are rare earth metal oxide material of the lanthanide elements, which have excellent biocompatibility and unique antioxidant capacity^13–15^. They are used in catalytic agents, metal-polishing agents, gas transducers, UV-screening agents, solar batteries, and solid oxide fuel cells^16–19^. The antioxidant properties of CeO_2_ NP have been attributed to the co-existence of two valence states in CeO_2_ (Ce^3+^ and Ce^4+^), with circular redox reactions occurring between these two oxidation states^20,21^. In these redox cycles, Ce^4+^ reverts to Ce^3+^ to leave equal numbers of oxygen vacancies as compensation. It has been revealed recently that the defect concentration at the CeO_2_ surface increases upon exposure to water, which could be relevant in living systems^22^. Recent studies have shown that CeO_2_ NP can protect neurocytes^14,23,24^ and myocardial cells^25,26^ against ROS damage by scavenging free radicals^15^. Genchi *et al*. found that CeO_2_ NP could potentially protect rat muscle cells against oxidative stress associated with microgravity and cosmic radiation by modulating gene expression^27^. Furthermore, CeO_2_ NP can suppress inflammation^28^ and diseases related to responses to oxidative stress, such as obesity^29^.

In the present study, we wished to ascertain if CeO_2_ NP could inhibit skin photoageing by clearing intracellular ROS. Hence, we proposed to build a model of skin photoageing by irradiating human skin fibroblasts with UVA *in vitro*. We evaluated the physical and chemical properties of CeO_2_ NP, their ability to participate in antioxidant defence, and the potential mechanism of action. Our data demonstrated the applications of CeO_2_ NP in research on the oxidative stress-related damage of skin photoageing and its potential prevention, and suggested that CeO_2_ NP could act as excellent photoprotective ingredients in cosmetics.

## Results

### Characterization of CeO_2_ NP

**Figure 1:**
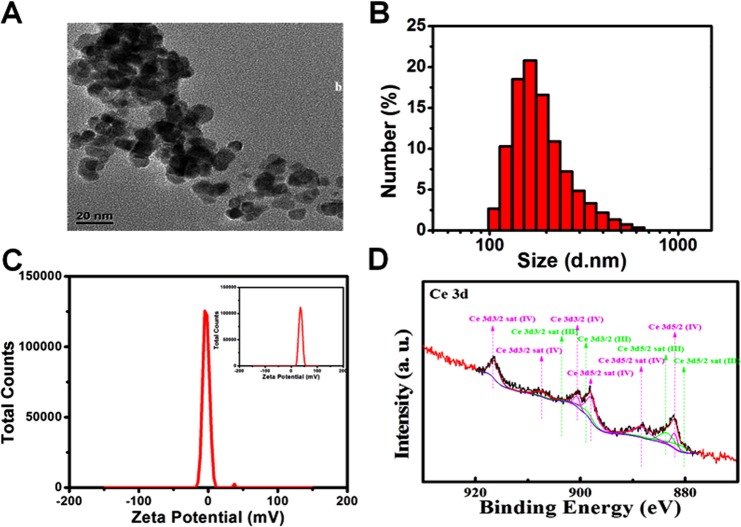
Characterization of CeO_2_ NP. (**A**) TEM image of CeO_2_ NP. The scale bar represents 20 nm. (**B**) The size distribution of CeO_2_ NP by DLS. (**C**) The zeta potential of CeO_2_ NP coated with BSA, and (inset) the zeta potential of CeO_2_ NP without BSA. (**D**) XPS spectrum of CeO_2_ NP showing a mixed valence (Ce^3+^ and Ce^4+^) state.

The morphology and size of CeO_2_ NP were determined using transmission electron microscopy (TEM). TEM images indicated that primary CeO_2_ NP appeared to be near spherical rather than polyhedral with regular morphology, and they had an aspect ratio close to 1 with uniform sizes of 10 ± 2.0 nm (Fig. 1A). However, NP were not negatively dyed with phosphotungstic acid, so BSA on the surface of NP could not be observed by TEM, which may cause the size of CeO_2_ NP to appear smaller than their actual size. Dynamic light scattering (DLS) was used to observe the hydrodynamic size distribution and zeta potential of CeO_2_ NP. The hydrated particle size of NP was ~197.6 nm, which have could have been due to slight agglomeration of NP and the presence of a hydration layer on their surface (Fig. 1B). The zeta potential of bare CeO_2_ NP and CeO_2_ NP with a BSA coating was about 35.7 mV and −2.87 mV, respectively, showing that modification of BSA reduced the positive charge of NP (Fig. 1C). X-ray photoelectron spectroscopy (XPS) revealed that CeO_2_ NP consisted of a mixture of Ce^3+^ and Ce^4+^ species (Fig. 1D). The Ce^3+^/Ce^4+^ ratio is 0.80 in the mixture, with the proportions of Ce^3+^ and Ce^4+^ being 44.56% and 55.44% respectively.

### Effect of exposure of CeO2 NP on HSFs viability

**Figure 2:**
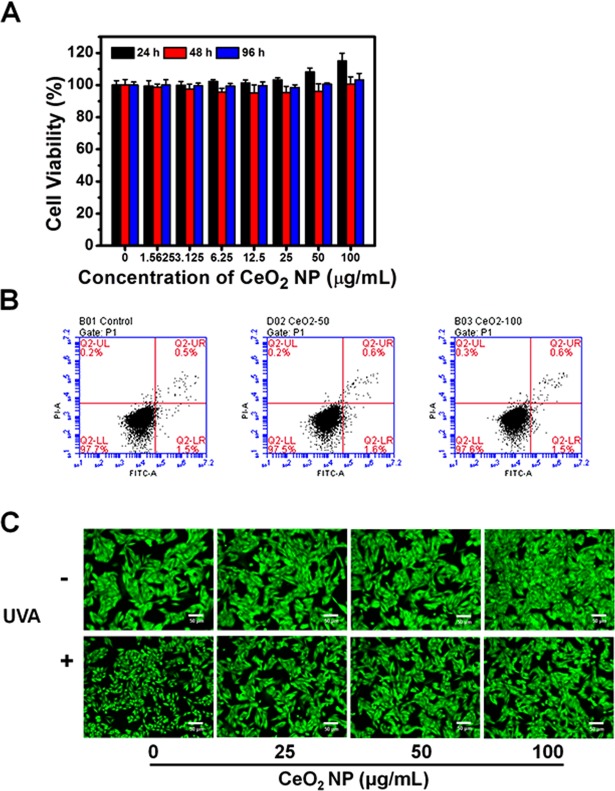
Effects of treatment by CeO_2_ NP on the viability and apoptosis of HSFs. (**A**) HSFs were treated with different concentrations of CeO_2_ NP for 24, 48 and 96 h, respectively. Then, HSF cell viability was evaluated by the CCK-8 assay. (**B**) Flow cytometric profiles of Annexin V-FITC/PI -stained apoptotic cells treated with CeO_2_ NP. (**C**) Fluorescence images using Calcein-AM staining. The scale bars in these images represent 50 µm.

Cell viability using a Cell Counting Kit-8 (CCK-8) kit revealed that CeO_2_ NP did not cause cytotoxicity after exposure for 24, 48 or 96 h, even at a high concentration of 100 μg/mL (Fig. 2A). The Annexin-V-FITC/PI assay was carried out to measure apoptosis after CeO_2_ NP treatment: significant apoptosis was not observed in any group (Fig. 2B). These results suggested their excellent biocompatibility and the possibility of a huge variety of biologic applications (e.g., nano-sized medicine carriers for targeted therapies).

Calcein-AM can pass through intact cell membranes and stay in the cytoplasm, where it is hydrolyzed into calcein by esterases, thereby emitting strong green fluorescence. In this assay, all HSFs were stained green and surviving cells were round after UVA irradiation alone (Fig. 2C), which is a typical change in cell morphology during apoptosis. However, in the CeO_2_ NP group, cells were viable with normal morphology in accordance with cells in the control group. These findings suggested the photoprotective effects of CeO_2_ NP against the cell injury induced by UVA irradiation.

### Effect of CeO_2_ NP on UVA radiation-induced SA-β-gal activity in HSFs

**Figure 3:**
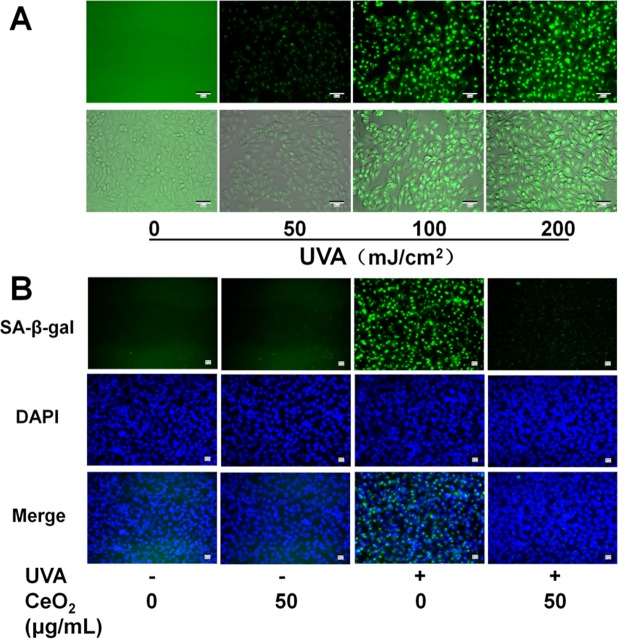
Effect of CeO_2_ NP on SA-β-gal activity in HSFs. (**A**) HSFs were irradiated by serial doses of UVA (50, 100, or 200 mJ/cm^2^) and stained with SA-β-gal. Scale bar = 50 μm. (**B**) Representative fluorescence images of cell nuclei stained with DAPI (blue) and SA-β-gal (green) in cells pretreated or not with CeO_2_ NP before exposure (or not) to UVA radiation (100 mJ/cm^2^). Scale bar = 20 μm.

The activity of SA-β-gal, biomarker of skin ageing, was evaluated by SA-β-gal staining assay carried out as mentioned previously^30–32^. SA-β-gal activity was enhanced with increasing doses of UVA irradiation (Fig. 3A). We chose a moderate intensity (100 mJ/cm^2^) for UVA exposure in subsequent studies based on Fig. 3A. CeO_2_ NP attenuated SA-β-gal activity in HSFs exposed to UVA radiation (100 mJ/cm^2^) clearly compared with treatment with UV radiation alone (Fig. 3B). These results demonstrated photoageing suppression by CeO_2_ NP.

### CeO_2_ NP suppress UVA radiation-induced ROS generation in HSFs

To confirm that CeO_2_ NP attenuated SA-β-gal activity in HSFs after exposure to UVA radiation by ROS scavenging, we measured intracellular ROS production using 2′,7′-dichlorofluorescein diacetate (DCFH-DA; Life Technologies, Carlsbad, CA, USA), a ROS fluorescent probe that emits green fluorescence when oxidized by ROS.

**Figure 4:**
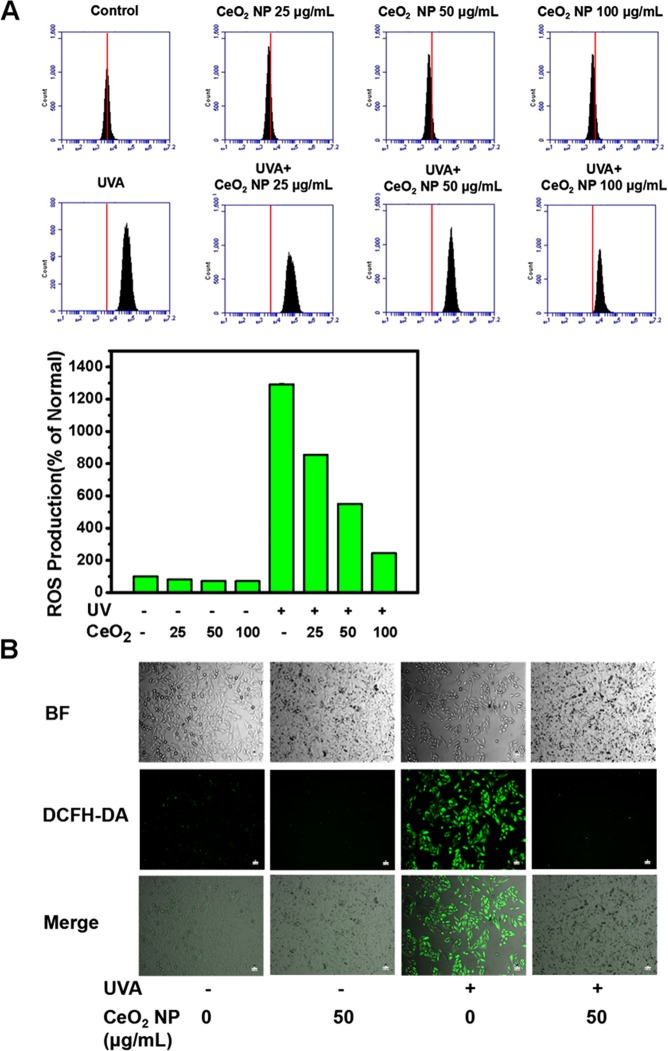
CeO_2_ NP suppress intracellular ROS production induced by UVA radiation. HSFs were treated, washed, and stained as mentioned in the Materials and Methods section. ROS levels in HSF cells were measured by a flow cytometer (**A**) and an inverted fluorescence microscope (**B**) labelled with DCFH-DA for 30 min at 37 °C. Scale bar = 10 µm.

A very weak fluorescence signal was observed in untreated cells and cells exposed only to CeO_2_ NP, whereas a much stronger fluorescence signal was observed after UVA irradiation (100 mJ/cm^2^) (Fig. 4B). Furthermore, the bright fluorescence signal was inhibited remarkably when CeO_2_ NP (50 μg/mL) were added (Fig. 4B). The results of quantitative measurements were consistent with the findings mentioned above (Fig. 4A). Several studies have shown, in accordance with our findings, that CeO_2_ NP can scavenge free radicals^24,26,33^.

### CeO_2_ NP protect HSFs from producing inflammatory factors and matrix-metalloproteinase (MMP)-2 induced by exposure to UVA radiation

IL-6, IL-8 and MMPs are the most prominent biomarkers of cell ageing^34^. Quantitative real-time quantitative-polymerase chain reaction (qRT-PCR) and ELISAs were applied to measure expression of IL-6, IL-8, and MMP-2 at gene and protein levels, respectively.

**Figure 5:**
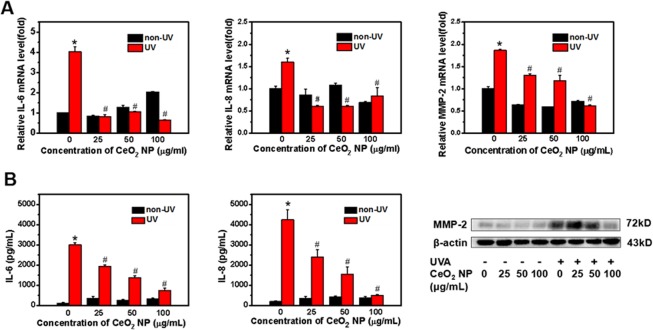
Biomarker expression in photoaged cells at gene and protein levels. (**A**) Relative mRNA expression of IL-6, IL-8 and MMP-2 compared with untreated cells. Data are represented as fold-changes. GAPDH was the internal reference gene in RT-PCR. (**B**) Concentrations of IL-6 and IL-8 in culture supernatants collected after exposure were examined using ELISA kits. Protein expression of MMP-2 after exposure to CeO_2_ NP or UVA radiation was determined by western blotting. β-Actin was the loading control for western blotting. Data are representative of three separate experiments. Data are the mean ± SD. *p < 0.05, compared with the control group. ^#^p < 0.05, compared with the group treated only with UVA radiation.

Relative RNA expression and concentrations of IL-6, IL-8 and MMP-2 in culture supernatants were increased significantly after exposure to UVA radiation compared with untreated cells (P < 0.05; Fig. 5). The presence of CeO_2_ NP led to suppression of the production of IL-6, IL-8, and MMP-2 upon simultaneous exposure to UVA radiation (Fig. 5). We did not observe dose-dependent protective effects of CeO_2_ NP on HSFs upon exposure to UV radiation.

### CeO2 NP inhibited UVA radiation-stimulated activation of c-Jun N-terminal kinases (JNKs) in HSFs

The mitogen-activated protein kinase (MAPK) signal-transduction pathway has a critical role in UV radiation-stimulated pathways, and modulates a sequence of downstream responses in human skin cells^35^. We examined the potential signalling pathways in UV radiation-induced skin ageing.

**Figure 6:**
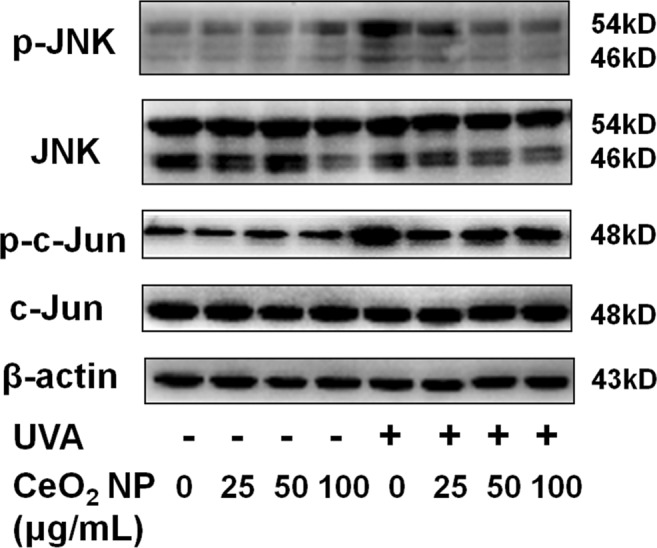
Western blotting of proteins to show the photoageing mechanism. HSFs were pretreated with (or without) CeO_2_ NP (25, 50 or 100 μg/mL), and irradiated (or not) by UVA. β-actin was the loading control for these analyses.

The phosphorylation of JNKs and c-Jun was induced by exposure to UVA radiation (Fig. 6), and then resulted in increased production of MMP-2 through a series of reactions. Addition of CeO_2_ NP reduced the expression of phosphorylated-JNKs, phosphorylated-c-Jun, and MMP-2 generation on account of UVA irradiation.

## Discussion

Over recent decades, nanotechnology has attracted considerable attention, and has been applied to biomedical applications such as treatment of cancer and photoageing^36^. Among these nanomaterials, CeO_2_ NP have been reported to exhibit antioxidant effects by scavenging free radicals in cells, and exerting catalytic effects by mimicking superoxide dismutase (SOD) and catalase activities^12,37^. The redox properties and toxicity of CeO_2_ NP are affected by their size, morphology, surface chemistry, and other factors, such as additives that coat the surface, local pH, and ligands that can participate in redox reactions^38,39^. CeO_2_ NP can internalize in human and animal cell lines and tissues and then localize with mitochondria, lysosomes and endoplasmic reticula as well as being abundant in the cytoplasm and the nucleus, thereby imparting protection against various oxidants^40,41^. The catalytic activity of CeO_2_ NP is dependent upon the surface Ce^3+^/Ce^4+^ oxidation state. CeO_2_ NP with a higher Ce^3+^ to Ce^4+^ ratio show higher SOD mimetic activity, and are more effective against oxidative stress-associated diseases or inflammation. NP with a lower Ce^3+^/Ce^4+^ ratio display higher catalase mimetic activity and possess anticancer or antibacterial activity^41,42^. Some studies have revealed that co-doped CeO_2_ NP can be used as clinical contrast agents for imaging and as therapeutic agents for cancer^43,44^. Prevention and treatment of skin photoageing have been the focus of scholarly research in recent years^45^. Nanomaterials are being used in cosmetics and skin-care products, especially NP of titanium oxide and zinc oxide. However, these NP have been reported recently to cause inflammation followed by ROS production, which can induce considerable damage to DNA^46,47^. In addition, reports have shown that these NP cannot reach the deeper stratum through the cuticle of the skin^48^. Therefore, we need try to find an efficient NP. Reports have shown that CeO_2_ NP can serve as effective radioprotectants for normal tissues, imparting protection against ROS^49,50^. Due to these characteristics, CeO_2_ NP have been used to protect against laser-induced retinal damage to reduce chronic inflammation^51,52^. Nevertheless, whether CeO_2_ NP can mitigate cellular senescence triggered by UV irradiation is not known. The present study was designed to verify the protective properties of CeO_2_ NP from UVA radiation-induced photoageing in HSFs, and postulated the potential signalling pathways involved.

Excessive production of ROS such as hydrogen peroxide, singlet oxygen, and hydroxyl radicals has been found to cause DNA injury and peroxidation of proteins and lipids, and can result in cancer, neurodegenerative disorders, or premature senility^37^. Studies have revealed a strong relationship between UV radiation and ROS generation^53,54^. Constant irradiation with UV light can give rise to inflammatory responses, break intracellular oxidation–reduction equilibria, contributing to ROS accumulation and, consequently, photoageing. Our findings suggest that exposure to UVA radiation could increase ROS generation dramatically, and that addition of CeO_2_ NP inhibited UVA radiation-stimulated overexpression of ROS in a dose-dependent manner (Fig. 4). The results of our studies are consistent with those mentioned above.

Cutaneous ageing involves reduced levels of mature collagen and enhanced expression of MMPs as well as degradation of proteins such as collagens, elastin, proteoglycans and fibronectin in the extracellular matrix^55–57^. MMP-1 breaks down collagen initially, and collagen is broken down further by MMP-2 and 9, which are crucial participators in the intrinsic and extrinsic ageing (photoageing) of skin^55,56^. We analyzed MMP-2 expression at gene and protein levels (Fig. 5). MMP-2 expression showed a dose-dependent decrease when HSFs were pretreated with CeO_2_ NP and exposed to UVA radiation (Fig. 5). We also evaluated SA-β-gal activity and secretion of cytokines such as interleukin IL-6 and IL-8, which are representative biomarkers of skin photoageing (Fig. 5). Our results indicated that expression of these markers, as a result of exposure to UVA radiation, was reduced by CeO_2_ NP pretreatment. We investigated UVA radiation-stimulated signal-transduction pathways by western blotting. A large body of evidence describes the role of the MAPK signalling pathway in UV radiation-activated skin damage. According to those studies, the increased production of ROS owing to UV radiation induces activation of the MAPK signalling pathway, which comprises JNKs, p38, MAPK, and extracellular signal-regulated kinases (ERKs)^53^. Subsequently, activator protein-1 (a heterodimer of c-Fos and c-Jun) and nuclear factor-kappa B are activated to modulate cellular proliferation and differentiation as well as inflammation and vasculogenesis^57,58^. In our study, ROS accumulation induced by UVA irradiation caused the phosphorylation of JNKs and c-Jun, resulting in incremental production of IL-6, IL-8 and MMP-2 through a series of reactions. Addition of CeO_2_ NP reduced the expression of phosphorylated-JNKs, phosphorylated-c-Jun, and IL-6, as well as the generation of IL-8 and MMP-2 resulting from UVA irradiation (Figs 5, 6).

Based on our findings, we hypothesize that CeO_2_ NP have great potential against UVA radiation-induced photoageing in HSFs because they can inhibit oxidative stress and DNA damage *via* regulation of the JNKs signal-transduction pathway. CeO_2_ NP could be used as photoprotective agents in the manufacture of cosmetics, applied in treatment of oxidative stress-associated diseases and prevention of skin photoageing.

## Methods

### Characterization of CeO_2_ NP

CeO_2_ NP were obtained from the Key Laboratory for the Biomedical Effects of Nanomaterials and Nanosafety within the National Center for Nanoscience and Technology of China (Chinese Academy of Sciences, Beijing, China). Analyses of morphology and size were undertaken using a transmission electron microscope (G-20; FEI, Hilsboro, OR, USA) at an operating voltage of 200 kV. The hydrodynamic size and zeta potential of CeO_2_ NP were measured by DLS using a ZetaSizer Nano ZS (Malvern Instruments, Malvern, UK) at room temperature. XPS was used to identify the valence state of Ce^3+^ and Ce^4+^.

### Cell culture

A HSF line was purchased from the Cell Bank of the Chinese Academy of Sciences (Shanghai, China). Cells were grown in Dulbecco’s modified Eagle’s medium (Gibco, Grand Island, NY, USA) supplemented with 10% foetal bovine serum (FBS, Gibco) and 1% penicillin/streptomycin (HyClone, Jülich, Germany) in a humidified atmosphere with 5% CO2 at 37 °C. In most studies, HSFs were starved upon reaching 85–90% confluence and used within passages 2–5.

### CeO_2_ NP treatment and exposure to UVA radiation

In experiments involving exposure to UV radiation, HSFs were pretreated with CeO_2_ NP dispersed in FBS-free medium for 24 h. Next, the suspensions of CeO_2_ NP were discarded, and cells were washed twice using phosphate-buffered saline (PBS) before exposure to ultraviolet radiation. Then, HSFs were maintained with a thin layer of PBS and irradiated with UVA (100 mJ/cm^2^). An ultraviolet lamp (peak, 365 nm; Vilber Lourmat, Marne-la-Vallée, France) delivered uniform radiation at 10 cm. After exposure to UVA radiation, HSFs were incubated with FBS-free media for an additional time according to the requirements of subsequent experiments.

### Cell-viability assays

To evaluate the viability of HSFs after exposure to CeO_2_ NP, we used a CCK-8 kit (Dojindo Laboratories, Kumamoto, Japan) for quantitative analyses and a LIVE/DEAD Cell Double Staining kit (Sigma-Aldrich) for qualitative analyses.

For the CCK-8 assay, HSFs were seeded in 96-well plates and incubated until they reached 70–75% confluence. Next, they were exposed to CeO_2_ NP (0, 1.5625, 3.125, 6.25, 12.5, 25, 50 or 100 μg/mL) for 24, 48 or 96 h, respectively. After exposure, PBS was used to wash cells twice. Then, 100 µL of CCK-8 solution was added to each well and incubated for an additional 1 h in an incubator. The absorbance of each well at 450 nm was measured by a microplate reader (Multiskan; Thermo Scientific, Waltham, MA, USA) after incubation.

In the calcein-AM staining assay, we seeded HSFs in 24-well plates and cultured them for 24 h. Then, HSFs were pretreated with fresh medium or CeO_2_ NP, before exposure (or no exposure) to UVA radiation. After treatments, HSFs were washed twice with PBS and stained by probes for 15 min. Fluorescence images were recorded using an inverted luminescence microscope (X73; Olympus, Tokyo, Japan) after being washed thrice with PBS.

### Measurement of intracellular ROS production

ROS production in HSFs was assessed using the fluorescence probe DCFH-DA (Life Technologies). HSFs were grown in six-well plates and then the two groups were exposed to CeO_2_ NP for 24 h. After cells had been washed with PBS and labelled with 20 µM of DCFH-DA for 30 min at 37 °C in an incubator in the dark, they were washed thrice and irradiated by UVA (100 mJ/cm^2^). Six-hours later, HSFs were collected by centrifugation (2000 rpm, 5 min, 4 °C) followed by detection using a flow cytometer at an excitation wavelength of 488 nm and emission wavelength of 530 nm. Intracellular ROS levels were in proportion to a mean fluorescence signal intensity of 10,000 HSFs.

An inverted fluorescence microscope was used to document fluorescence images for qualitative investigations after cells had been cultured in 24-well plates and treated, washed, and stained as mentioned above.

### qRT-PCR

HSFs were cultured in six-well plates and treated with CeO_2_ NP or UVA irradiation (100 mJ/cm^2^). Then, total RNA was extracted using TRIzol^®^ Reagent (Sigma–Aldrich) following manufacturer instructions. A reverse-transcriptase system (Promega, Fitchburg, WI, USA) using 2 μg of total RNA was employed. A real-time PCR instrument (Realplex4; Eppendorf, Hamburg, Germany) was used for PCR amplification. Each sample was assayed in triplicate.

The primer sequences (forward and reverse, respectively) were: 5′-TGAGAACGGGAAGCTTGTCA-3′ and 5′-ATCGCCCCACTTGATTTTGG-3′ for glyceraldehyde 3-phosphate dehydrogenase; 5′-GGACTTAGACCGCTTGGCTT-3′ and 5′-GTGTTCAGGTATTGCATGTGCT-3′ for MMP-2; 5′-GGATTCAATGAGGAGACTTGCC-3′ and 5′-TGGCATTTGTGGTTGGGTCA-3′ for IL-6; 5′-CACCGGAAGGAACCATCTCA-3′ and 5′-TGGCAAAACTGCACCTTCACA-3′ for IL-8. All of primers were designed by Primer Premier 5.0.

### Determination of cytokine levels using ELISAs

HSFs were grown in 24-well plates, pretreated with CeO_2_ NP for 24 h, and exposed to UVA radiation. Then, culture supernatants were collected and examined using human IL-6 and IL-8 ELISA kits (R&D Systems, Minneapolis, MN, USA) according to manufacturer instructions. Concentrations of IL-6 and IL-8 were obtained using standard curves.

### Senescence-associated SA-β-gal activity

Measurement of SA-β-gal activity was done by SA-β-gal staining using a Senescence β-galactosidase staining kit (Cell Signalling Technology, Danvers, MA, USA). First, HSFs were cultivated in 24-well plates, pretreated with CeO_2_ NP, and exposed to UVA radiation. After washing with PBS, 3% formaldehyde was employed to fix cells for 5 min at room temperature. Next, HSFs were washed before incubation with a SA-β-gal probe for 6 h. Images were obtained by a fluorescence inverted microscope.

### Western blotting

HSFs were cultured, treated with CeO_2_ NP before exposure to UVA radiation as stated above, and washed twice with cold PBS. RIPA lysis buffer with the addition of protease inhibitors (1 mM of phenylmethane sulfonyl fluoride) was applied to HSFs lysing on ice. After centrifugation (14,000 g, 10 min, 4 °C), the total protein concentration was quantified using a Bicinchoninic Acid Protein Assay kit (Thermo Scientific). Proteins were separated by 10% (*w/v*) sodium dodecyl sulfate–polyacrylamide gel electrophoresis, electrotransferred onto polyvinylidene difluoride (PVDF) membranes, and blocked with 5% skimmed-milk powder. After PVDF membranes had been labelled with various primary antibodies overnight at 4 °C, they were incubated with secondary antibody for 1 h at room temperature. The signal visualization of proteins was carried out by an Odyssey Infrared Imaging system (Li-Cor Biosciences, Lincoln, NB, USA).

### Statistical analyses

Results are the mean ± standard deviation. Statistical analyses were done using the Student’s t-test or one-way ANOVA using SPSS v20 (IBM, Armonk, NY, USA). P < 0.05 was considered significant.
